# Evaluation of the Respiratory Microbiome and the Use of Tracheal Lavage as a Diagnostic Tool in Kemp’s Ridley Sea Turtles (*Lepidochelys kempii*)

**DOI:** 10.3390/ani11102927

**Published:** 2021-10-10

**Authors:** Kerry L. McNally, Jennifer L. Bowen, Jennifer O. Brisson, Adam Kennedy, Charles J. Innis

**Affiliations:** 1 Animal Health Department, New England Aquarium, Boston, MA 02110, USA; cinnis@neaq.org; 2 Marine Science Center, Department of Marine and Environmental Sciences, Northeastern University, Nahant, MA 01908, USA; je.bowen@northeastern.edu; 3 Massachusetts Veterinary Referral Hospital, Ethos Veterinary Health, Woburn, MA 01801, USA; jbrisson@ethosvet.com; 4 Rescue & Rehabilitation Department, New England Aquarium, Boston, MA 02110, USA; akennedy@neaq.org

**Keywords:** *Lepidochelys kempii*, pneumonia, microbiome, tracheal lavage

## Abstract

**Simple Summary:**

A tracheal lavage is commonly used to characterize the microbes that may be causing pneumonia in sea turtles, typically by culture-dependent methods. In this study, we characterized the tracheal lavage microbiome through culture-independent methods and compared the resulting sequence data to conventional cultures, the degree of radiographic lung abnormalities, and pathogens of sea turtles as previously reported in the literature. This study also evaluates the microbial communities at different sections of the respiratory tract from deceased sea turtles. We found that radiographic lung abnormalities do not correlate with the tracheal lavage microbiome, tracheal lavage cultures under-represent the microbial community as determined by culture-independent methods, many previously reported sea turtle pathogens are present in low abundance of the tracheal lavage microbiome, and tracheal lavages are not representative of other sections of the respiratory tract.

**Abstract:**

Respiratory disease is a common cause of morbidity and mortality in sea turtles, including the Kemp’s ridley sea turtle (*Lepidochelys kempii*). Although culture-dependent methods are typically used to characterize microbes associated with pneumonia and to determine treatment, culture-independent methods can provide a deeper understanding of the respiratory microbial communities and lead to a more accurate diagnosis. In this study, we characterized the tracheal lavage microbiome from cold-stunned Kemp’s ridley sea turtles at three time points during rehabilitation (intake, rehabilitation, and convalescence) by analyzing the 16S rRNA gene collected from tracheal lavage samples. We retrospectively developed a radiographic scoring system to grade the severity of lung abnormalities in these turtles and found no differences in diversity or composition of microbial communities based on radiographic score. We also found that the culture isolates from tracheal lavage samples, as well as other previously reported sea turtle pathogens, were present in variable abundance across sequenced samples. In addition to the tracheal microbial community of live turtles, we characterized microbial communities from other segments of the respiratory tract (glottis, trachea, anterior lung, posterior lung) from deceased turtles. We found a high degree of variability within turtles and a high degree of dissimilarity between different segments of the respiratory tract and the tracheal lavage collected from the same turtle. In summary, we found that the pulmonary microbial community associated with pneumonia in sea turtles is complex and does not correlate well with the microbial community as identified by tracheal lavage. These results underscore the limitations of using tracheal lavage for identification of the causative agents of pneumonia in sea turtles.

## 1. Introduction

Respiratory abnormalities are prevalent in sea turtles, including Kemp’s ridley sea turtles (*Lepidochelys kempii*), and are a common cause for their morbidity and mortality [1]. Initial diagnosis of pneumonia typically relies on radiographic evidence of lung abnormalities by identifying patterns of radiopacities, which may increase with inflammation, edema, and fibrosis [1,2]. Abnormalities found on radiographs that typically indicate pneumonia can include focal, multifocal, and generalized interstitial patterns to a reticular (honeycomb) pattern [2], all of which help veterinarians identify the severity and location of infection. Veterinary staff use a variety of diagnostic methods to further characterize and identify the causative microbes (bacteria and/or fungi) to determine the appropriate course of medical intervention.

Tracheal lavage, or “tracheal wash”, is a diagnostic tool that is commonly used in animals, including sea turtles, for characterizing pneumonia via cytology and culture [1,3]. The tracheal lavage process involves infusing a limited volume of sterile saline into the trachea (typically 0.5% to 1.0% of the body weight, although lesser volume is common and adequate). The saline contacts the biofilm and epithelial cells of the respiratory tract such that when it is aspirated, it can be cultured to identify potential causative microbes [1]. Culture results from tracheal lavage samples guide treatment based on the bacteria and/or fungi that are isolated and associated antibiotic susceptibility testing. Although diagnosis via this method can result in successful rehabilitation, we do not know whether tracheal lavage results are truly representative of the microbes that are responsible for causing pneumonia. More thoroughly characterizing the microbial communities of the respiratory system may help us to better understand the causes of respiratory disease, as well as the reliability of common diagnostic methods such as tracheal lavage in sea turtles.

Research on the human respiratory microbiome over the past decade recognizes the importance of microbial communities in health and chronic respiratory diseases, including pneumonia [4,5]. Microbial communities that inhabit healthy individuals become altered in taxonomic identity, diversity, and richness as healthy lungs transition to various disease states [6]. Several factors influence the lung microbiome, including microbes immigrating to the lungs through aspiration or inhalation, microbial emigration through host immune defenses, and growth conditions that influence community members, such as temperature [6,7,8,9]. Respiratory disease can alter the composition of the lung, and the microbial community structure in diseased lungs is more highly variable than in healthy individuals [6,10]. Changes in respiratory microbial communities, and the interaction of microbes along the respiratory tract can influence the complex pathogenesis of diseases such as pneumonia [11]. Respiratory microbiome research focusing on non-human vertebrates and their diseases is also important yet limited. Identifying potential pathogens and important commensal species in these complex systems can lead to a better understanding of diseases and treatment options.

There are limitations in the use of culture-dependent methods for diagnosis of pneumonia [11]. Less than 1% of bacteria in nature can be cultured or grown in media [12,13] due to a lack of knowledge of the specific growth requirements, as well as difficulty in replicating environmental conditions in the laboratory [13]. This is true for host-associated microbes as well. Standard medical culture media only identifies 70% of bacteria in the human body, leading to difficulty in diagnosing potential pathogens [4]. For example, 75% of humans diagnosed with pneumonia have no specific pathogen identified, creating difficulty in treating the infection when drug sensitivity cannot be ascertained [4]. In dogs, the causes of pneumonia are better understood through culture-independent methods versus standard culture, with sequencing of the bronchoalveolar lavage demonstrating distinct signatures, such as overgrowth of a single microbe, not identified in culture samples [14].

Different diagnostic tools will provide different results for both culture-dependent and culture-independent methods. In cattle, tracheal lavage samples had drastic differences in cytological findings compared to bronchoalveolar lavage due to the location of the respiratory tract sampled (trachea vs. bronchioles), and tracheal lavages had higher nasopharyngeal contamination [15]. Further, cultures of deep oral swabs are not appropriate surrogates to tracheal lavage samples in dogs, as each resulted in different isolates [16]. In sheep, there is spatial variation among the lung microbial communities, with microbiome differences based on depth in the respiratory tract, or distance from the glottis [17]. This regionality of the respiratory tract microbiome is likely found in other species.

In this study, we investigated the respiratory microbiome of cold-stunned, or hypothermic, Kemp’s ridley sea turtles with and without lung abnormalities throughout the duration of their rehabilitation. Kemp’s ridley sea turtles, a critically endangered species, become stranded annually in Cape Cod Bay, Massachusetts, United States of America, from cold-stunning when water temperatures drop during autumn [18,19,20,21]. When found alive, they are admitted to wildlife rehabilitation hospitals such as the New England Aquarium (NEAq) for triage and rehabilitation with the goal of returning healthy animals back to the wild. Cold-stunned Kemp’s ridley sea turtles present with a wide range of pathologic findings including cardiorespiratory depression, dehydration, sepsis, reduced renal function, and death [22,23,24,25,26,27,28]. Pneumonia is extremely common, with an average of half of the Kemp’s ridley sea turtles per cold-stun event presenting with respiratory abnormalities during rehabilitation at NEAq [2,24].

The first objective of this study was to characterize the turtles’ tracheal lavage microbiome. We hypothesized that turtles with lung abnormalities (i.e., pneumonia) would have a distinct microbial community in tracheal lavage samples from turtles with no lung abnormalities. Through a retrospective radiographic review, we developed a radiographic scoring system to grade the degree of lung abnormalities to further test this hypothesis. The conventional view in veterinary medicine is that an overgrowth of a pathogenic bacteria causes infection; thus, we hypothesized that the microbial community of turtles that present with signs of pneumonia have a lower overall diversity due to a higher abundance of one or a few specific bacteria associated with the disease. Additionally, we investigated whether different conclusions about animal health can be drawn from diagnosis based on tracheal lavage cultures compared to high throughput sequencing of the microbial community. We hypothesized that the culture results of tracheal lavages were not truly capturing potential causative agents for pneumonia in turtles. We also identified the prevalence of previously reported sea turtle pathogens in the tracheal lavage sequence dataset. Using culture swabs, we characterized microbial communities in other locations along the respiratory tract of deceased cold-stunned turtles and compared them to the tracheal lavage fluid from the same turtles. We hypothesized that the tracheal lavage microbiome was similar to that of the tracheal swab microbiome, but distinct from the lung microbiomes. Therefore, tracheal lavage may not be representative of the lower respiratory tract where infections can occur; thus, it may not be the most valuable diagnostic tool in characterizing pneumonia in sea turtles.

## 2. Materials and Methods

### 2.1. Sample Collection

Kemp’s ridley sea turtles were admitted to NEAq during the 2015 cold-stun event (November and December 2015). We chose turtles at random to have radiographs taken on the day of admission to assess for lung abnormalities. Radiographic results dictated whether the turtle was enrolled in the study, with the goal of having an approximately equal number of turtles with radiographically normal lungs and those with evidence of pneumonia. One of three attending veterinarians categorized the turtle as having pneumonia or not having pneumonia (hereafter referred to as “non-pneumonia”) based on their interpretation of the initial radiographs. This allowed the veterinarians to assess the radiographs in real time and treat the patient accordingly.

We conducted tracheal lavages on the day of admission (“intake”) without sedation, as turtles were hypothermic and minimally reactive. Tracheal lavages were performed using the following process. First, we intubated the trachea of the turtle with a sterile Cole-style endotracheal tube (Jorgensen Labs, Loveland, CO, USA) that was selected based on the size of the turtle. Then, we inserted a sterile 5 French (1.7 mm) diameter red rubber catheter as far as possible into the endotracheal tube. We infused five mL of sterile saline through the catheter while the turtle was gently rocked side to side to promote fluid contact with the lung tissue. We then aspirated the saline with a syringe, repeating as needed to recover a range of two to four mL. If an animal was classified as a pneumonia turtle, we aliquoted a portion of the fluid and debris into three empty sterile vials. If an animal was classified as a non-pneumonia turtle, we saved two vials. For pneumonia turtles, one vial was refrigerated and submitted for aerobic, anaerobic, fungal, and mycobacterial cultures to a commercial veterinary diagnostic laboratory (IDEXX Laboratories, North Grafton, MA, USA) within eight hours of collection. We immediately placed the remaining vials of tracheal lavage material on dry ice after collection and moved them to an ultra-low freezer (−80 °C) within 15 min of collection for DNA extraction and sequencing at a later date.

NEAq veterinarians prescribed antibiotics for the turtles as necessary based on radiographic findings and blood analysis. The most common drugs used initially were ceftazidime (22 mg/kg intramuscularly every 3 days) or oxytetracyline (42 mg/kg subcutaneously every 6 days). Treatment was adjusted as needed based on clinical response and culture results of the tracheal lavage. We sampled surviving turtles at two additional time points during the rehabilitation process. The “rehab” sample was collected approximately eight weeks after admission but, in certain cases, was conducted as early as six weeks after admission to ensure that the sample was collected prior to the end of antibiotic use. The “convalescent” sample was collected when the turtle was classified by the attending veterinarian as clinically healthy (based on appetite, physical exam, serial blood data, radiographs, etc.), approximately 30 days after antibiotics were discontinued, if antibiotics had been used. If a turtle was not on antibiotics, convalescence was determined based on the veterinarian’s evaluation of clinical status and ability to be released (dependent on appetite, physical exam, and fitness for transport). At each of these time points, we repeated a tracheal lavage, sedating the turtles to ensure safe restraint. For sedation, we administered 0.1 mg/kg dexmedetomidine (Dexdomitor^®^, Zoetis, Parsipanny, NJ, USA) intravenously and allowed it to take effect for approximately 10 min before the tracheal lavage was performed. Heart rate and palpebral reflex were monitored throughout the process. Once the tracheal lavage was completed, we reversed the sedative by administering 1.0 mg/kg atipamezole (Antisedan^®^, Zoetis, Parsipanny, NJ, USA) intramuscularly.

We performed necropsies on eight turtles, four of which were already enrolled in the study and received tracheal lavages while alive, and four of which had not been previously enrolled in the study but were utilized opportunistically. Necropsies were performed within 12 h of death. During the time of necropsy, we examined all organ systems. We performed a post-mortem tracheal lavage if it had not been done within the previous two days. We used sterile cotton tipped applicators to swab other portions of the respiratory tract which included the glottis, the trachea (cranial to the bifurcation), the anterior right lung, and the posterior right lung. We placed swabs and tracheal lavage fluid in labelled cryovials and immediately stored them on dry ice until moving them to a −80 °C freezer within 15 min of collection for later DNA extraction and sequencing. We collected a swab of visible respiratory lesions using the Fisherfinest^®^ Transport Swab with Amies gel (Fisher HealthCare, Pittsburg, PA, USA) for culture submission to IDEXX Laboratories. We also collected a set of tissues, including lung and trachea, in 10% neutral buffered formalin that we submitted for histopathology to National Marine Fisheries Service Office of Protected Resources Pathology Consultation at University of Florida, Gainesville, FL, USA.

### 2.2. Culture Methods

Cultures were performed by IDEXX Laboratories as previously described [29]. Isolates were identified by matrix-assisted laser desorption-ionization time-of-flight mass spectrometry (Bruker Scientific LLC, Billerica, MA, USA). Antimicrobial susceptibility was determined via commercially available VITEK Colorimeter susceptibility cards (bioMerieux Inc., Durham, NC, USA) and Kirby–Bauer disk diffusion assay in accordance with performance standards of the Clinical and Laboratory Standards Institute [30]. Selection of antimicrobials for susceptibility testing was determined by the diagnostic laboratory. Minimal inhibitory concentrations were determined, and isolates were classified as susceptible, intermediate, or resistant.

### 2.3. Retrospective Radiographic Review and Scoring System

In addition to initial radiographs, we obtained radiographs of the turtles throughout rehabilitation using a standardized method, including a dorsoventral and craniocaudal horizontal beam view via a veterinary radiographic system (MinXray HF100+, MinXray Inc., Northbrook, IL, USA) with digital X-ray cassettes (Kodak DirectView CR cassette, Carestream Health Inc., Rochester, NY, USA) at a focal distance of 1 m. Typical exposure factors for both projections were 75 kVp and 7.5 mAs. A single, board-certified veterinary radiologist, blinded to the clinical status of the turtles, retrospectively reviewed the radiographs obtained closest to the time of each tracheal lavage and scored the pulmonary changes according to the grading scale detailed in Table 1. Additionally, the radiologist further categorized radiographic lung abnormalities according to distribution (left, right, bilateral, diffuse, or ventral).

### 2.4. DNA Extraction and Sequencing

We extracted DNA from necropsy swabs and tracheal lavage fluid using a phenol:chloroform:isoamyl extraction protocol adapted from Mettel et al. [31]. First, we vortexed and centrifuged the tracheal lavage fluid or swabs in PBL lysis buffer (water saturated phenol, disodium EDTA, sodium dodecyl sulfate, Tris HCL, pH 5.7). The supernatant was removed and placed in a clean tube. We then added TPM buffer (50 mM Tris, pH 7.0, polyvinyl pyrrolidone, and MgCl_2_) to the original tube after the supernatant was removed. The supernatant created after vortexing and centrifuging was added to the tube with the first supernatant. We then added 800 μL of a phenol:chloroform:isoamyl alcohol solution (pH 6.7+, 25:24:1) to the combined supernatant. After centrifuging, the upper aqueous layer was transferred to a sterile tube. To this tube, we added 0.7 volume of 100% isopropanol and 0.1 volume of 3 M sodium acetate. The supernatant was decanted after centrifugation and the remaining pellet rinsed with 70% ethanol and allowed to air dry. We used 50 μL nuclease-free water to resuspend the dried pellet. Since we were working with low biomass samples, we proceeded with a clean-up protocol to concentrate the DNA. This included adding 0.1 volume 3 M sodium acetate and 2 volumes of 100% isopropanol to the suspended pellet, vortexing briefly, and then freezing the sample for 15 min. We then centrifuged the sample, removed the supernatant, and washed the pellet with 70% ethanol, decanted, and centrifuged again. We allowed the pellet to air dry, and then resuspended it in 12 μL nuclease-free water. Once we verified all DNA extracts by gel electrophoresis, we stored it at −80 °C until amplification. To ensure there was no contamination from supplies or solutions used in the extraction, we also verified negative controls of sterile saline and unused sterile swabs.

We amplified the DNA extracts in triplicate using bacterial specific (515F and 806R), uniquely barcoded, 16S rRNA primers containing adaptors for Illumina sequencing [32]. Each 25 μL PCR reaction contained 12.5 μL Phusion Master Mix (ThermoFisher, Waltham, MA, USA), 0.5 μL primers, 10 μL diethylpyrocarbonate (DEPC) water, and 2 μL of DNA. After we verified the PCR product via gel electrophoresis, the PCR product was then purified using AMPure XP beads (Beckman Coulter, Inc. Indianapolis, IN, USA) following manufacturers guidelines and using the 0.8:1.0 ratio of bead:sample to target 300 bp and above. We then quantified the DNA using the Agilent D1000 ScreenTape System (Agilent Technologies, Inc., Waldbronn, Germany) following manufacturers guidelines. We pooled the purified PCR product to equimolar concentrations. To ensure proper DNA size selection from the pool, we size selected the product using a BluePippin™ (Sage Science Inc., Beverly, MA, USA) following manufacturer’s instructions. Sequencing was performed on the Illumina MiSeq platform with a paired-end V2 500 cycle kit (Illumina Inc., San Diego, CA, USA).

Raw sequence data are available at NCBI’s Sequence Read Archive, under BioProject accession number: PRJNA760794.

### 2.5. Data Analysis

We used Illumina-utils version 2.0.2 [33] to demultiplex paired-end reads. We used DADA2 version 1.12.1 [34] in R version 3.6.1 [35] to perform quality filtering. This included the removal of chimeras, singletons, chloroplasts, mitochondrial DNA, and archaea. DADA2 was also used for the merging of paired reads and amplicon sequence variant (ASV) clustering. Taxonomy was assigned using IDTAXA from the DECPHER package version 2.12.0 [36] with the Silva Small Subunit (SSU) 132 training set for classification. We used the phyloseq package version 1.28.0 in R to further process the sequences, evaluate taxonomy, and perform diversity metric visualizations and statistical tests [37].

We used Bray–Curtis distance metrics to analyze microbial community differences in tracheal lavage samples and used principal coordinates analysis (PCoA) to visualize these differences. We tested for significant differences using pairwise permutational multivariate analysis of variance (PERMANOVA) for variables including timepoint, initial radiographic scores, disease condition (non-pneumonia or pneumonia) and survival with *p* values adjusted for multiple comparisons using the Benjamini–Hochberg procedure. Shannon diversity was calculated for survival, disease condition, timepoint, initial radiographic score, and initial radiographic abnormality locations and patterns. Since the data were not normally distributed (Shapiro–Wilk, *p* = 0.024), we tested for significance using pairwise Wilcoxon tests. A *p* value of <0.05 was considered statistically significant following application of the Benjamini–Hochberg procedure.

We compiled tracheal culture results from IDEXX Laboratories and identified the isolates grown in culture at the different sampling times and the percentage of antibiotic sensitivity for those isolates. We then identified ASVs in the corresponding sequence data that had the same genus as the isolates and performed a BLASTN [38] search to determine the closest bacterial species. The ASVs were then tracked through the time course of the animal’s rehabilitation. We screened the tracheal lavage microbiome sequences from admission for the presence of potential pathogens common to sea turtles at the genus and family level based on pathogens as summarized by Innis and Frasca [39]. To further characterize the respiratory tract, we visualized microbial community composition of necropsy samples using taxa plots for each sample to identify the individual turtle’s variability. We used Bray–Curtis distance metrics to determine the similarity of the tracheal lavage microbial community to other sites along the respiratory tract (oral/glottis, trachea, anterior lung, and posterior lung). We also reviewed and summarized the histopathology reports for the turtles that had tissues submitted.

## 3. Results

### 3.1. Sample Data

We collected tracheal lavage samples for sequencing from 20 turtles on the day of admission, 10 of which were classified as non-pneumonia and 10 as pneumonia. Six of these turtles died during rehabilitation (prior to “rehab” sample collection), and of these, we necropsied four. Necropsies were not performed on the other two mortalities due to use of the carcasses for an unrelated project. We collected additional tracheal lavage samples from eleven turtles in rehabilitation (mean 48 days in rehabilitation, range 30–58 days) and from fourteen of the turtles at convalescence (mean 100 days in rehabilitation, range 24–201 days) since three of the turtles were considered convalescent before their rehab sample could be collected.

Out of 45 tracheal lavage samples, sequencing of the 16S rRNA gene resulted in 718,312 reads after joining paired-end reads and quality filtering. The mean sequence counts per sample was 16,325 (median 13,752) and range was 267 to 42,742 counts per sample. These sequences were assigned to 1331 unique ASVs across 218 different families. Negative control samples had no reads remaining after quality filtering and were thus not included in any sequence analysis.

**Figure 1:**
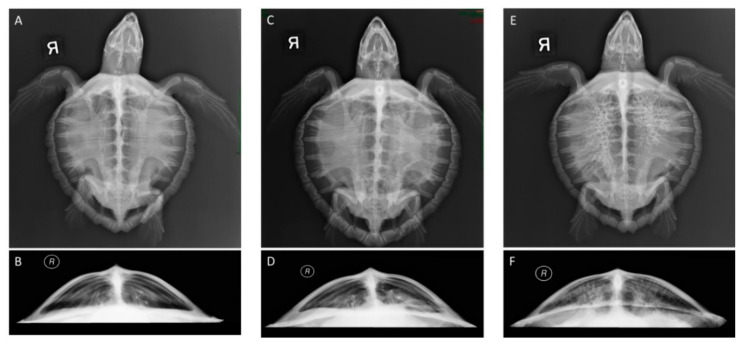
Examples of radiographs from Kemp’s ridley sea turtles with different radiographic scores (Table 1, Table S1). (**A**) Dorsoventral view of a normal turtle (Score 0); (**B**) craniocaudal view of a normal turtle (Score 0); (**C**) dorsoventral view and (**D**) craniocaudal view of Score 2 with abnormalities in the ventral left lung; (**E**) dorsoventral view and (**F**) cranioventral view of Score 5, most severe with diffuse reticular (honeycomb) pattern in both lungs, worse in right lung.

Radiographic scores were assigned to 22 turtles at admission retrospectively. These included the 14 surviving turtles and eight necropsied turtles. Fifteen turtles had bilateral lung abnormalities at admission, four of which were more severe on the right lung and one that was more severe on the left lung. Only five turtles had completely unilateral abnormalities, four on the right lung only and one on the left lung only. Approximately half of the turtles with lung abnormalities had diffuse patterns. The radiologist noted a reticular pattern in seven turtles, primarily associated with a radiographic score of 5, and four of these turtles died in rehabilitation. Examples of score 0 (normal lungs), score 2 (mild abnormalities), and score 5 (severe abnormalities) are provided in Figure 1. Additional scores were assigned to 11 rehab turtles and the 14 convalescent turtles. No turtles had a score of 5 at convalescence, although one turtle still had a radiographic score of 4 based on remaining lung abnormalities despite being clinically healthy. Table S1 provides detailed information on radiographic scores assigned to each turtle as well as the location and patterns described.

We collected 38 samples at necropsy from eight turtles, which included four body sites (glottis/oral, trachea, anterior right lung, and posterior right lung) and tracheal lavages for all except two turtles who had tracheal lavages performed within the previous 48 h (Turtle ID 56 and 68). From these 38 necropsy samples, sequencing of the 16S rRNA gene resulted in 980,793 reads after joining paired-end reads and quality filtering. One sample (Turtle ID 27 anterior lung) had low sequence depth and was removed during quality filtering. The mean sequence count per sample was 27,244 (median 12,215) and range was 463 to 183,855 counts per sample. These sequences were assigned to 1179 unique ASVs.

### 3.2. The Tracheal Lavage Microbiome

**Figure 2:**
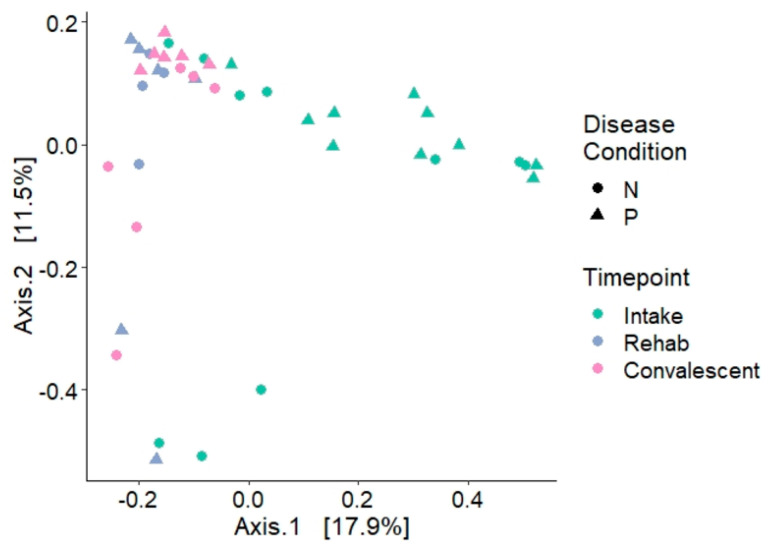
Principal coordinate analysis (PCoA) plot of Bray–Curtis distance for tracheal lavage samples of Kemp’s ridley turtles collected throughout rehabilitation for cold-stunning. Each color indicates the timepoint during hospitalization and each shape indicates whether the turtle was categorized as a non-pneumonia (N) or pneumonia (P) patient based on initial radiographs. Intake samples were significantly different from rehab and convalescent samples (PERMANOVA, *p* = 0.001). Rehab and convalescent samples were not significantly different from each other (PERMANOVA, *p* = 0.227). There were no significant differences due to disease condition at any timepoint.

The tracheal lavage of cold-stunned Kemp’s ridley sea turtles at admission was significantly different from other time points during rehabilitation based on Bray–Curtis distance metrics (Figure 2, PERMANOVA, *p* = 0.001). The rehab and convalescent tracheal lavage results, however, were not significantly different from each other (PERMANOVA, *p* = 0.227). There was no difference in Shannon diversity among all time points (Wilcoxon, *p* > 0.05). At admission, Shannon diversity of tracheal lavage samples was not significantly different as a function of survival of the turtles (those that survived vs. those that died), disease condition (non-pneumonia vs. pneumonia), radiographic scores, location of radiographic abnormalities, or radiographic abnormality patterns (Wilcoxon, *p* > 0.05). Bray–Curtis distance was also not significantly different between these variables (PERMANOVA, *p* > 0.05).

**Figure 3:**
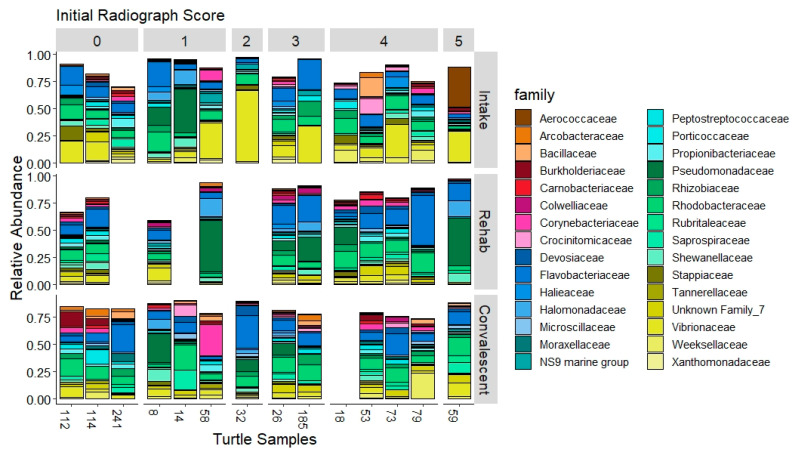
Taxa plots representing the relative abundance of the top 30 families across samples of cold-stunned Kemp’s ridley turtles that survived. Plots are separated by radiographic score at time of intake (0 through 5) and timepoint during hospitalization. A turtle has no sample during rehab if it was considered convalescent before a sample could be collected. Any other samples that are missing are due to low quality sequencing results and were removed during quality filtering.

Bacterial families prevalent in 100% of the tracheal lavages at admission include *Vibrionaceae*, with a mean of 21.1% relative abundance, *Rhodobacteraceae* (9.3%), and *Flavobacteriaceae* (8.8%). *Vibrionaceae*, although present in all the samples, had high variability with a range of 0.2% to 70.0% relative abundance across samples. *Shewanellaceae* was also highly variable with a mean of 3.7% and range of 0.03% to 32.0%. *Pseudomonadaceae* was present in 85% of the tracheal lavage samples with a mean of 5.2% and range of 0% to 40% abundance (Figure 3).

In comparison to samples collected upon admission, rehabilitation and convalescent tracheal lavage microbial community composition had different proportions of the most abundant bacterial families (Figure 3). They had a lower abundance of *Vibrionaceae* than found in intake samples, with a mean of 3.8% (range 0–11.4%) in rehab samples and 4.8% (range 1.6–12.0%) in convalescent samples. Both time points were dominated by *Flavobacteriaceae* (rehab 15.8%, convalescent 12.2%) and *Rhodobacteraceae* (rehab 9.4%, convalescent 11.7%). *Pseudomonadaceae* remained highly variable in abundance at each of these time points (rehab mean 13.3%, range 0.5–47.5%; convalescent mean 4.2%, range 0–27.2%).

### 3.3. Comparison of Culture-Independent and Culture-Dependent Methods

We submitted seven tracheal lavage samples to IDEXX Laboratories for culture at each time point (total 21 samples). The laboratory reported positive cultures as presence of growth and, where possible, they reported the identification of the isolate. Anaerobic, mycobacterial, and fungal cultures had no growth. Aerobic cultures resulted in more isolates from the intake tracheal lavages and rehab samples compared to convalescent samples (Table 2). *Vibrio* sp. were cultured at all three time points from admission to convalescence, and not always from the same turtle. Most turtles typically had one to two isolates that were cultured per sample, with no growth on two turtles at intake, two during rehabilitation, and four at convalescence. Several cultures also grew non-enteric gram negative bacteria that could not be speciated. Specific species that were only isolated at admission were *Raoultella planticola* and *Shewanella putrefaciens*. *Pseudomonas* sp. and *Enterococcus* sp. were both only isolated from tracheal lavages of turtles in rehabilitation.

Drug sensitivities were also reported for each of the isolates, except for one *Enterococcus* sp., which did not receive a complete panel. All isolates were sensitive to several antibiotics, including amikacin, ciprofloxacin, enrofloxacin, gentamycin, tetracycline, and tobramycin. Isolates had the highest percentage of resistance to amoxicillin and cephalexin. All isolates except *Vibrio* sp. were completely sensitive to ceftazidime, but some *Vibrio* isolates had intermediate resistance (Table S2).

**Figure 4:**
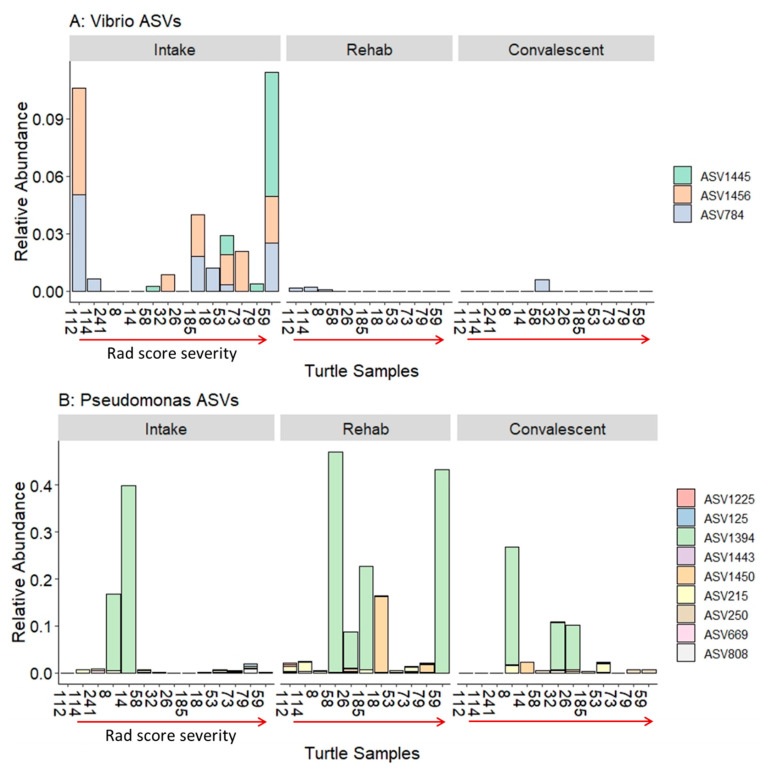

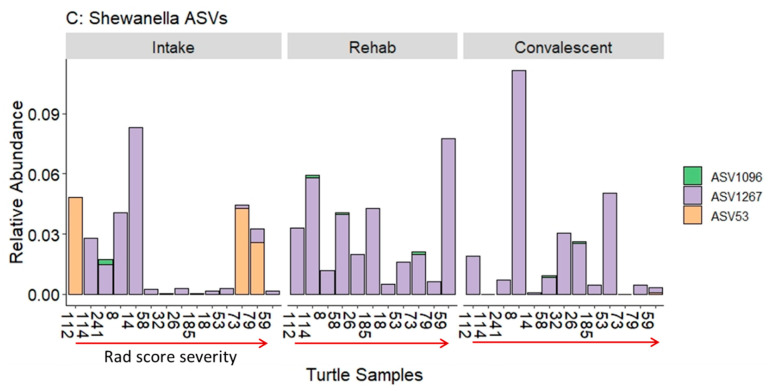
Bar plots of the relative abundance (y axis) of ASVs in the sequence dataset that match the genus level of tracheal lavage culture results. The genera represented are *Vibrio* (**A**), *Pseudomonas* (**B**), and *Shewanella* (**C**). The turtle samples (Turtle ID on the x axis) are ordered by their intake radiographic score from 0 (normal) on the left to 5 (severe) on the right separated into facets for each timepoint (intake, rehab, convalescent).

To compare results from the culture analysis to our sequencing results, we examined ASVs that were identified as belonging to the same genus as those found in culture. Culture isolates that matched at the genus level in the sequence data include *Vibrio* (3 ASVs), *Pseudomonas* (9 ASVs), and *Shewanella* (3 ASVs). The BLAST matches of these ASVs resulted in up to three possible species based on 100% identity across the length of the 16S rRNA fragment (Table 3). The *Vibrio* ASVs had highest abundance in the intake samples and lower abundance in samples in rehabilitation and at convalescence (Figure 4A). *Pseudomonas* had variable abundance throughout rehabilitation, with ASV1394 (*Pseudomonas flourescens*) having greater than 40% relative abundance in some samples (Figure 4B). *Shewanella woodyi* (ASV53) had high abundance in some intake tracheal lavages, while *Shewanella algae* (ASV1267) was prevalent in most tracheal lavages throughout rehabilitation (Figure 4C). None of the genera were specific to higher radiographic scores as they were variable across samples with pneumonia (Figure 4). The patterns observed in *Vibrio* and *Shewanella* ASV abundance did not parallel their observed importance in the culture analysis. *Vibrio* was cultured during rehabilitation and convalescence while it was predominantly found in intake samples from the sequence dataset (Table 2, Figure 4A). Conversely, *Shewanella* was only cultured in one intake sample, while the sequence data shows *Shewanella* across all timepoints (Table 2, Figure 4C).

### 3.4. Previously Reported Sea Turtle Pathogens in Sequence Data

We found several previously reported sea turtle pathogens in low abundance at the genus level in our tracheal lavage microbial communities including *Corynebacterium*, *Flavobacterium*, *Acinetobacter*, and *Mycobacterium*, meanwhile the potential pathogens *Pseudomonas* sp., *Shewanella* sp. and *Vibrio* sp. were in higher abundance at the genus level (Table 4). Since taxonomic identification did not reach the genus level for the majority of the ASVs, we found that at the family level, there was high abundance of *Flavobacteriaceae* (8.82%) and *Vibrionaceae* (21.14%) across intake tracheal lavages with 48 ASVs and 12 ASVs, respectively (Table 4). *Pseudomonadaceae* and *Shewanellaceae* had similar prevalence at the genus level but with 8 and 4 ASVs, respectively.

### 3.5. Necropsy Samples

**Figure 5:**
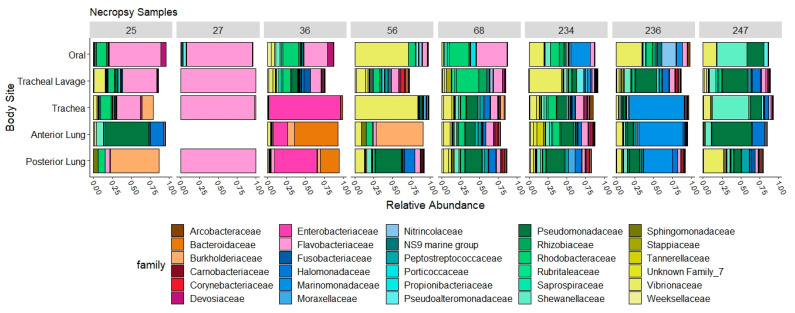
Taxa plots representing the relative abundance (x axis) of the top 30 families in necropsy samples of Kemp’s ridley sea turtles at each site of the respiratory system, including a tracheal lavage. Plots are separated by individual turtles (Turtle ID numbers are listed at the top).

**Figure 6:**
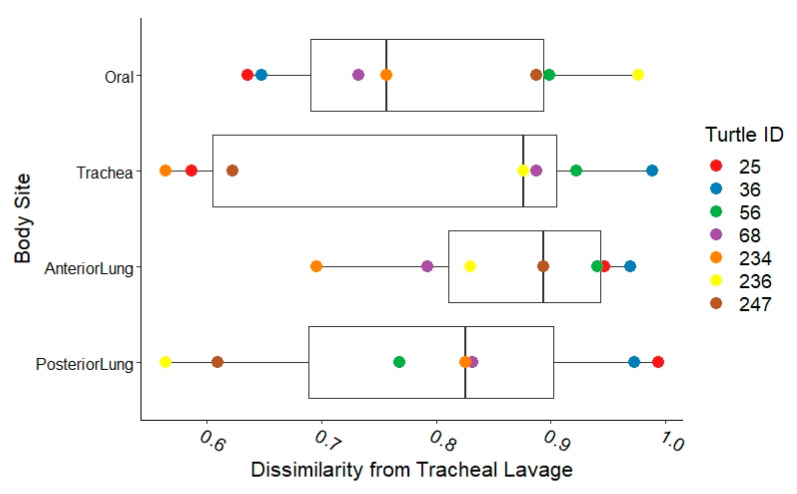
Boxplots of Bray–Curtis dissimilarity of post-mortem sequences along the respiratory tract of Kemp’s ridley sea turtles in comparison to the corresponding individual’s tracheal lavage sequences. The higher the Bray–Curtis value (x axis), the more dissimilar the sample is from the corresponding tracheal lavage. Colored points represent the Turtle ID number.

From eight necropsies, we found high variability in the abundance of taxa at each body site, with some turtles having high abundance (greater than 40%) of *Flavobacteriaceae*, *Enterobacteriaceae*, *Marinomonadaceae*, *Burkholderiaceae*, *Bacteroidaceae*, *Pseudomonadaceae*, *Shewanellaceae*, or *Vibrionaceae* in the trachea and/or lung samples (Figure 5). Due to the variability between turtles, we only compared Bray–Curtis similarity among the body sites of each turtle to the corresponding tracheal lavage of that turtle (that is, we only performed intra-turtle comparisons, not comparisons across individuals) and found that most samples were distinctly different from the tracheal lavage microbial community (Figure 6).

Histologic evaluation confirmed the diagnosis of pneumonia for all four turtles for which necropsy was completed (Table 5). Three out of the four turtles had granulomatous pneumonia with intralesional fungi +/− bacteria, and the fourth turtle had intralesional bacteria. The tracheal lavage cultured isolates did not correspond to the cultured isolates from necropsy samples of the lungs, though they were collected up to 4 weeks prior to necropsy (Table 5). Fungi were cultured from two out of three necropsy lung samples, despite no positive fungal cultures from tracheal lavages at admission.

## 4. Discussion

### 4.1. The Respiratory Microbiome and Pneumonia

Respiratory diseases such as pneumonia are typically associated with a higher bacterial biomass, decreased bacterial community diversity, and a shift in composition to a higher abundance of single pathogens [5,6,7,11,14,40]. In this study, characterization of tracheal lavage microbial communities did not identify any of these typical signatures of pneumonia. We found no correlation between bacterial diversity nor composition with disease severity at the time of admission.

The respiratory tract is a heterogenous ecosystem with a continuous mucosal surface and a continuously varying microbial topography [4]. Even in healthy lungs of humans, the microbial communities are not consistent across samples [41]. The lung microbiome of mice is also highly variable and clusters by different habitats in the respiratory system [42]. Similarly, the upper respiratory tract of tortoises does not have a strong core nasal microbiome but does share microbiomes based on habitat [43]. Similar to mammals, the local environment likely influences the respiratory microbiome of sea turtles through microbial immigration, such as microaspiration, inhalation of microbes, and dispersal along mucosal surfaces, and elimination, including exhalation and removal via host immune defenses [4,5,11]. Further, in this study, cold-stunned sea turtles all stranded in different locations in Cape Cod Bay, allowing for potential geographic variation in microbial exposure. Turtles stranded in various states of debilitation, with potential variation in the amount of sea water aspirated, respiratory rates, immunologic status, and other physical and physiologic conditions that are not necessarily associated with radiographic lung abnormalities. Thus, it is possible that cold-stunning further complicates the respiratory microbiome. Pneumonia leads to a disruption in homeostasis of the immigration and elimination of microbes that can occur from changing conditions such as the environment of the lung (i.e., systemic vasoconstriction from hypothermia, hypoxia), host inflammatory response (i.e., immunocompromised state from cold-stunning), and interactions with other bacteria that may change from aspiration/introduction of new microbes or environmental conditions [5].

### 4.2. The Tracheal Lavage Microbiome over Time

Changing conditions during rehabilitation likely caused the shift in tracheal lavage microbial communities after admission, since the water temperature and local environment (e.g., tank water) became consistent, and turtles were medically treated for cold-stun clinical signs (dehydration, metabolic derangements, etc.). Concurrent with this shift in environment once in rehabilitation, the microbial community composition also stabilized. Temporal stability in bottlenose dolphin blowhole microbial communities also showed host specificity, where the samples of individual dolphins were more similar over time than to other dolphins [44]. Although overall communities were not different between rehab and convalescent samples, variability remained within the individual turtles, which was different than the pattern observed in dolphins [44].

### 4.3. The Tracheal Lavage Microbiome and Degree of Radiographic Lung Abnormalities

The radiographic scoring system was useful in identifying the most critically ill turtles retrospectively. Further categorizing the scores into unilateral or bilateral, and ventral or diffuse provided insight into potential causes of pneumonia in cold-stunned sea turtles. Potential causes include colonization of opportunistic microbes and/or aspiration of sea water [2,23]. Although we primarily saw bilateral lung abnormalities, the right lung tended to be more severely affected radiographically than the left as described in a previous study [2]. If aspiration of sea water contributes to pneumonia in these cases, it seems reasonable that the right lung would be more severely affected since the entrance to the right bronchus is ventral to the entrance of the left bronchus anatomically, allowing water and debris to gravitate in that direction [2]. Radiographic score did not correlate with survival, similar to other studies [2], but we did see that the reticular pattern was associated with the highest radiographic scores. This reticular pattern corresponds to the structure of the airways and is possibly representative of edicular wall thickening.

There was good agreement in interpretation of initial radiographs between attending veterinarians (who interpreted the radiographs in real time) and the veterinary radiologist (who interpreted radiographs retrospectively), with non-pneumonia turtles generally having lower radiographic scores, and pneumonia turtles having higher radiographic scores (Table S1). However, agreement was imperfect for some cases (e.g., a turtle was categorized as non-pneumonia but had a radiographic score of 3). There are many possible reasons for such discrepancies including lack of standardized objective radiographic evaluation methods for chelonian lungs, subjectivity of the evaluator, differences in experience, knowledge, and skill between general clinicians and radiologists, and the use of binary categorization (clinicians) vs. a five-point scale (radiologist). It is possible that the scoring system used here may be useful for future evaluation of sea turtle lungs.

We found that most turtle radiographic lung abnormalities resolved or improved by convalescence, but there were still four that had persistent moderate abnormalities. These turtles were still considered clinically stable and ultimately released. Since it is difficult to radiographically distinguish chronic pneumonia (infection still present) from fibrotic changes (infection resolved, but residual scarring present) clinicians often rely on the total body of clinical information to determine convalescence and suitability for release to the wild, sometimes pursuing lung biopsy for definitive diagnosis of questionable cases [1]

### 4.4. Culture-Dependent vs. Culture-Independent Methods

Comparison of culture-dependent and culture-independent methods highlights the limitations of culture in diagnosing diseases such as pneumonia. We found that some cultured isolates were not present in the sequences or possibly not detectable due to extremely low abundance (*Raoultella planticola*, *Enterococcus* sp.). In addition, several samples had isolates that could not be speciated (reported as non-enteric gram negative rods); thus, we could not examine them in the sequence dataset. Of the species that were identified, *Vibrio* sp., *Pseudomonas* sp. and *Shewanella* sp. were all isolated at various time points, and they were also found in various abundances in the 16S rRNA sequences (Figure 4). *Vibrio* sp. are gram negative rods associated with the marine environment, yet some species have been found in septic sea turtles [45]. Tracheal lavage samples at admission had higher abundances of *Vibrio* overall than rehab and convalescent samples. This could indicate influence of environment or immune status during the rehabilitation process. One *Vibrio* (ASV1445) appeared to be more prevalent in some turtles with higher radiographic scores. The BLAST match of this ASV was *Vibrio anguillarum*, which is a known pathogen of marine fish, bivalves, and crustaceans, and thus may have pathogenicity in sea turtles as well [46]. *Pseudomonas* sp. were also found in a varying abundance across tracheal lavage samples. *Pseudomonas* are found in the marine environment, have extensive antimicrobial resistance profiles, and are opportunists [45]. The ASV with relatively high abundance in several samples was *Pseudomonas flourescens*, which produces antimicrobial metabolites that may be useful in defense roles [47]. Lastly, *Shewanella* sp., in particular, *Shewanella algae* was abundant in most tracheal lavage samples at each time point. This marine bacterium can tolerate a wide range of temperatures and salinity [48]. Although it has been shown to be a pathogenic agent [48], it was present in convalescent turtles; thus, it was unlikely to be pathogenic in this context.

The mismatch between culture and sequence data may result from several possibilities. Culture relies on morphological and biochemical identification which can lead to decreased specificity [13]. We saw this in the culture-based identification of “non-enteric gram negative rods”, and several isolates that were only identified to the genus level. Growth can also be limited by conditions of the media environment (temperature, pH, nutrients) or inhibition by other microbes [13,39,45]. A negative culture may reflect the presence of culture inhibitors such as *Pseudomonas flourescens*, which actively impedes in vitro growth of other microbes by producing antimicrobial metabolites [11,47]. Concurrent or recent antibiotic use could also inhibit bacterial viability in cultures. Lastly, some of the pathogens that may be causing pneumonia may be uncultured microbes that have not been previously identified [14]. Although cultures have limitations and 16S rRNA data provide a more comprehensive profile of bacterial taxa in samples, antibiotic susceptibility testing from cultured isolates does seem to provide clinical benefit in identifying sensitivity and resistance for drug selection [39]. It is possible that the antimicrobial profile of cultured isolates provides a reasonable proxy for the antimicrobial profile of the uncultured organisms. Unfortunately, it often remains unclear whether the cultured isolate is truly the causative agent for pneumonia.

### 4.5. Screening of Previously Reported Pathogens in the Microbiome

Despite the limitations in tracheal lavage samples as a diagnostic tool, we screened the sequences for previously reported pathogens of sea turtles and found low abundance of several pathogens at the genus level and high abundance in some bacteria at the family level (Table 4). Although the known pathogen *Flavobacterium* sp. was in low abundance, sequence data showed 48 ASVs in the corresponding family, *Flavobacteriaceae*. *Flavobacteriaceae* is common in marine environments [49], which likely explains the high number of ASVs contributing to the family. *Pseudomonas*, *Shewanella*, and *Vibrio* were identified in low abundance at the genus level, but *Vibrio* at the family level (*Vibrionaceae*) was found in high abundance likely due to its association with the marine environment and not necessarily due to its pathogenicity in tracheal lavages. Many potential pathogens appear in low abundance in healthy animals, but they could lead to secondary invasion in immunocompromised states [5,14,43]. Since detection of sea turtle pathogens has primarily relied on culture-dependent methods, potential pathogens are not limited to those in Table 4 and it is expected that many more pathogens could be captured with higher taxonomic resolution of microbial community analysis.

### 4.6. Regionality of the Respiratory Tract Microbiome

Analysis of necropsy tissue microbial communities not only revealed high variability between individual turtles, but also between sites along the respiratory tract (Figure 5). Lung brush samples in sheep found similar patterns, in that multiple samples along the respiratory tract were more similar in the same sheep than between samples of different sheep, yet spatial variability was still present [17]. The variability along the respiratory tract may be due to regional changes of physiological parameters, such as gas concentrations, osmolarity, temperature, pH, and blood flow [5,17,50,51]. These differences demonstrate that certain parts of the respiratory tract cannot be considered surrogates for other parts. For example, oral microbial communities were found to provide different culture results than tracheal lavages in dogs with pneumonia, and thus could not be considered a replacement diagnostic tool [16]. We also found differences within the same lung of individual turtles; thus, dissimilarity of the tracheal lavage microbial communities to other parts of the respiratory tract was somewhat expected.

### 4.7. Tracheal Lavage as a Diagnostic Tool

Since tracheal lavage results were not similar to other sites of the respiratory tract (Figure 6), infection within the lung might not be characterized effectively by tracheal lavage. Tracheal lavage as performed in this study is a blind diagnostic technique, and the clinician does not know how deep into the respiratory tract the saline infuses, or whether the right side or left side of the tract is entered. Since lung abnormalities develop in different locations (i.e., unilateral vs. bilateral) and in different patterns within the lung (ventral vs. diffuse), failure to sample the infected region of the lung could lead to misleading results. Endoscopically guided lung lavage allows visualization of the lung, may provide guidance to the site of infection, and is recommended for cases of chronic or resistant pneumonia if resources are available [1]. Although performed as sterilely as possible, the oral cavity may contaminate tracheal lavage samples. For example, cattle had more evidence of oral microbial contamination in tracheal lavage samples compared to bronchoalveolar lavage [15]. In humans, bronchoalveolar lavage microbial communities were more similar to the mouth than lung brushings, which reflects the difference in sampled surface areas [42]. Brushings, which can be performed by bronchoscopy, may provide more thorough and representative samples by improved contact with the lung biofilm and epithelial cells since tracheal lavages potentially have a dilution effect from the saline and a lack of clarity around which surface area was actually sampled [11]. Histopathologic findings also highlight the limitations of tracheal lavages in that bacteria and/or fungi were typically intralesional. Tracheal lavages would not penetrate well encapsulated granulomas, and since granulomatous pneumonia was common in turtles with lung lesions (Table 5), biopsy may be a more valuable approach. Overall, we found that the use of tracheal lavage as a diagnostic tool was limited due to the wide differences in the microbial communities between the tracheal lavage microbial communities and those found in the necropsy samples.

## 5. Conclusions

We established that the lungs of Kemp’s ridley sea turtles are not sterile, but contain a diverse assortment of microbial taxa. Our data demonstrate that the microbial communities recovered from tracheal lavage samples are diverse and variable between cold-stunned sea turtles at stranding, rehabilitation, and convalescence. The radiographic scoring system identified severity of lung abnormalities; however, tracheal lavage microbial communities did not cluster by radiographic score, likely because pneumonia in sea turtles has complicated and highly variable causes. Bacteria isolated from culture-dependent methods, which in many cases could not be assigned taxonomically at the species level, had variable abundances in culture-independent methods. We found some cultured isolates in the sequence data across many samples, including at convalescence, and they were not necessarily dependent on radiographic severity. We documented several previously identified bacterial pathogens in varying abundances in the tracheal lavage microbial communities of both pneumonia and non-pneumonia turtles, suggesting that these organisms may be opportunistic pathogens that can be present in turtles that do not have pneumonia. We also found that tracheal lavage samples were not representative of other segments of the respiratory tract in sea turtles, which is likely due to a combination of regionality of the lungs, granulomatous lesions/focal sites of infection, and limitations of the technique itself (access to lungs, contamination, visualization, surface area sampled). Our findings suggest that tracheal lavage may not be the most definitive diagnostic tool for determining causative agents for treatment selection.

## Figures and Tables

**Table 1:** Radiographic scoring system established to identify degree of lung abnormalities for Kemp’s ridley sea turtles in this study.

Score	Description	Radiographic Features
0	Normal	No radiographic abnormalities of the pulmonary parenchyma
1	Minimal	Interstitial, increased opacity with ill-defined pulmonary vasculature
2	Mild	Diffuse interstitial
3	Mild-Moderate	Mixed interstitial and alveolar pattern, increased opacity with obscured pulmonary vasculature
4	Moderate	Alveolar focal or multifocal, mild reticular
5	Marked	Diffuse bilateral alveolar or marked reticular pattern

**Table 2:** Identity of the bacteria cultured from the tracheal lavage samples of cold-stunned Kemp’s ridley sea turtles classified as Pneumonia.

	Intake	Rehab	Conv
	*n*	*n*	*n*
*Vibrio alginolyticus*	1	1	0
*Pseudomonas* sp.	0	2	0
*Raoultella planticola*	1	0	0
*Vibrio* sp.	2	3	3
*Shewanella putrefaciens*	1	0	0
*Enterococcus* sp.	0	1	0
Non-enteric gram neg rod	3	2	1
No growth	2	2	4

*n* = number of samples from which the bacteria were isolated; Conv, convalescent.

**Table 3:** Taxonomy of ASVs from the sequence dataset matching at the genus-level to tracheal lavage culture results. Sequences of each ASV were input into the NCBI BLASTN database. The top species level results (BLAST Matches column) are included, which match the ASV at 100% sequence identity across the length of the fragment.

ASV	Genus	BLAST Matches
ASV784	*Vibrio*	*chagasii*, *cuclitrophicus*, *splendidus*
ASV1445	*Vibrio*	*anguillarum*, *cortegadensis*
ASV1456	*Vibrio*	*tapetis*
ASV1225	*Pseudomonas*	*graminis*, *viridiflava*, *donghuensis*
ASV125	*Pseudomonas*	*marginalis*, *grimontii*, *rhodesiae*
ASV1394	*Pseudomonas*	*flourescens*, *veronii*
ASV1443	*Pseudomonas*	*koreensis*
ASV1450	*Pseudomonas*	*pachastrellae*, *aestusnigri*
ASV215	*Pseudomonas*	*oleovorans*, *stutzeri*, *taeanensis*
ASV250	*Pseudomonas*	*arsenicoxydans, yamanorum*, *proteolytica*
ASV669	*Pseudomonas*	*paralactis*, *tolaasii*, *gessardii*
ASV808	*Pseudomonas*	*entomophila, mosselii*, *guariconensis*
ASV1096	*Shewanella*	*marisflavi*, *fidelis*, *schlegeliana*
ASV1267	*Shewanella*	*algae*
ASV53	*Shewanella*	*woodyi*

**Table 4:** List of previously reported bacteria associated with infection in sea turtles [39] with % prevalence (relative abundance) in tracheal lavage samples from study turtles at intake. Prevalence is listed both at the genus level and family level with the number of ASVs composing each. SD, standard deviation.

Bacteria	Genus Prevalence %	Genus SD %	Number of ASVs in Genus	Family	Family Prevalence %	Family SD %	Number of ASVs in Family
*Aeromonas hydrophila*	0	0	0	*Aeromonadaceae*	0	0	0
*Bacillus* sp.	0	0	0	*Bacillaceae*	1.3	3.5	7
*Corynebacterium* sp.	1.7	2.1	8	*Corynebacteriaceae*	2.1	2.4	13
*Hafnia alvei*	0	0	0	*Enterobacteriaceae*	0.35	0.43	2
*Citrobacter braakii*	0	0	0	*Enterobacteriaceae*			
*Citrobacter freundii*	0	0	0	*Enterobacteriaceae*			
*Escherichia coli*	0	0	0	*Enterobacteriaceae*			
*Enterococcus* spp.	0	0	0	*Enterococcaceae*	0.09	0.16	1
*Flavobacterium* sp.	0.07	0.12	1	*Flavobacteriaceae*	8.82	6.45	48
*Acinetobacter calcoaceticus*	0.66	0.7	5	*Moraxellaceae*	1	0.9	10
*Chromobacterium violaceum*	0	0	0	*Neisseriaceae*	0.17	0.33	4
*Pseudomonas* sp.	5.10	12.3	7	*Pseudomonadaceae*	5.15	12.2	8
*Shewanella algae*	3.60	7.2	3	*Shewanellaceae*	3.66	7.14	4
*Vibrio alginolyticus*	2.10	3.0	4	*Vibrionaceae*	21.14	20.13	12
*Vibrio fluvialis*				*Vibrionaceae*			
*Vibrio cholerae*				*Vibrionaceae*			
*Mycobacterium* spp.	0.03	0.1	1	*Mycobacteriaceae*	0.03	0.1	1

**Table 5:** Table summarizing histopathologic results from four Kemp’s ridley sea turtles with corresponding lung culture results and tracheal lavage culture results from intake.

Turtle ID	Date of Admit	Date of Necropsy	Histologic Diagnoses (Lung)	Culture Isolates from Tracheal Wash at Admit	Culture Isolates at Necropsy
25	23 November 2015	14 December 2015	Severe necrotizing and granulomatous pneumonia with intralesional fungal hyphae (with vascular invasion)	*Serratia liquefaciens* *Shewanella* sp. *Vibrio* sp. Non-enteric gram neg rod	*Hafnia* (*Enterobacter*) *alvei* *Mucor* sp. *Paecilomyces* sp.
27	23 November 2015	2 December 2015	Heterophilic bronchopneumonia with intralesional bacteria	NE	NE
36	24 November 2015	26 Dececmber 2015	Severe necrotizing, heterophilic, and granulomatous pneumonia with numerous intralesional fungi and bacteria	*Vibrio* sp.	*Serratia marcesens* *Citrobacter braakii* *Providencia rettgeri*
247	20 December 2015	14 February 2016	Severe, diffuse, chronic granulomatous pneumonia with intralesional fungal hyphae	NE	gram neg rods (rare) * *Paecilomyces* sp. *

NE, not examined; * indicates that the culture of the lung was taken while the turtle was still alive (via a biopsy) prior to mortality.

## Data Availability

All sequencing data and metadata are available at NCBI’s Sequence Read Archive, under BioProject accession number: PRJNA760794.

## Supplementary Materials

The following are available online at https://www.mdpi.com/article/10.3390/ani11102927/s1, Table S1: Radiographic scores assigned to study turtles as described in Table 1; Table S2: Identity and percentage of antibiotic sensitivity for bacteria cultured from the tracheal wash samples of turtles classified as Pneumonia.
